# Self-Assembly of 1D Double-Chain and 3D Diamondoid Networks of Lanthanide Coordination Polymers through In Situ-Generated Ligands: High-Pressure CO_2_ Adsorption and Photoluminescence Properties

**DOI:** 10.3390/molecules26154428

**Published:** 2021-07-22

**Authors:** Chatphorn Theppitak, Suwadee Jiajaroen, Nucharee Chongboriboon, Songwuit Chanthee, Filip Kielar, Winya Dungkaew, Mongkol Sukwattanasinitt, Kittipong Chainok

**Affiliations:** 1 Thammasat University Research Unit in Multifunctional Crystalline Materials and Applications (TU-McMa), Faculty of Science and Technology, Thammasat University, Pathum Thani 12121, Thailand; chatphorn.the@dome.tu.ac.th (C.T.); suwadee.jia@dome.tu.ac.th (S.J.); nucharee.cho@dome.tu.ac.th (N.C.); songwuit.c@gmail.com (S.C.); 2 Department of Chemistry, Faculty of Science and Technology, Thammasat University, Pathum Thani 12121, Thailand; 3 Department of Chemistry, Faculty of Science, Naresuan University, Phitsanulok 65000, Thailand; filipk@nu.ac.th; 4 Department of Chemistry, Faculty of Science, Mahasarakham University, Maha Sarakham 44150, Thailand; winya.d@msu.ac.th; 5 Department of Chemistry, Faculty of Science, Chulalongkorn University, Bangkok 10330, Thailand; mongkol.s@chula.ac.th

**Keywords:** coordination polymers, CO_2_ adsorption, hydrazide, in situ synthesis, lanthanide, photoluminescence

## Abstract

Two new lanthanide-based coordination polymers, [Sm_2_(bzz)(ben)_6_(H_2_O)_3_]·0.5H_2_O (**1**) and [Eu(bbz)(ben)_3_] (**2**), were synthesized and characterized. The described products were formed from in situ-generated benzoate (ben) and *N*’-benzoylbenzohydrazide (bbz) ligands, which were the products of transformation of originally added benzhydrazide (bzz) under hydrothermal conditions. Compound **1** exhibits a one-dimensional (1D) double-chain structure built up from the connection of the central Sm^3+^ ions with a mixture of bzz and ben ligands. On the other hand, **2** features a 3D network with a 4-connected (6^6^) dia topology constructed from dinuclear [Eu_2_(ben)_6_] secondary building units and bbz linkers. High-pressure CO_2_ sorption studies of activated **1** show that maximum uptake increases to exceptionally high values of 376.7 cm^3^ g^−1^ (42.5 wt%) under a pressure of 50 bar at 298 K with good recyclability. Meanwhile, **2** shows a typical red emission in the solid state at room temperature with the decay lifetime of 1.2 ms.

## 1. Introduction

Coordination polymers are a class of crystalline organic-inorganic materials built by connecting metal ions or clusters and organic bridges through coordination bonds [1]. These hybrid solid materials possess the intriguing architectures of variable dimensionality that allow potential applications in the fields of adsorption, luminescence, catalysis, and magnetism [2,3,4,5,6]. Lanthanide-based coordination polymers (LnCPs) have drawn much attention during the past few decades due to their unique optical properties, such as high luminescence efficiency, long luminescence lifetimes, and narrow bandwidths, allowing for potential applications in sensing, lighting, and integrated optics [7]. In comparison with transition metal-based coordination polymers, the rational design and synthesis of LnCPs is more difficult since Ln^3+^ ions frequently show higher coordination numbers (typically from 6 to 12) and flexible coordination geometries. Moreover, various synthetic parameters, such as the ratio of reactants, solvent, reaction temperature, and pH can have an influence on the nucleation and crystal growth of the final products [8,9,10,11]. Therefore, experimental investigation of the impact of synthetic parameters on the crystallization process and self-assembly of LnCPs is still required. It is well known that Ln^3+^ ions have a high affinity for and prefer to bind to hard donor atoms. In this regard, oxygen- and/or nitrogen-containing organic ligands, in particular benzene- and pyridine carboxylates, have been widely used in the development of novel lanthanide-based luminescent materials. These types of ligands can adopt various coordination modes with respect to the central Ln^3+^ ions. They can also function as light-harvesting antennas for efficient sensitization of Ln^3+^ ions to enhance their emission intensity [12,13,14].

On the other hand, hydro (solvo) thermal reactions accompanied by in situ ligand synthesis have been developed as one of the most feasible strategies for the construction of novel coordination polymers [15]. For instance, a variety of intriguing structural topologies of tetrazole-based polymeric complexes have been successfully prepared through in situ [2 + 3] cycloaddition reaction of nitriles and an azide in the presence of metal salts under hydrothermal conditions [16,17]. Moreover, this synthetic approach can also be extended to a wide number of carboxylate-based coordination polymers derived from the hydrolysis of ester or cyano groups in water [18,19].

We are interested in the synthesis of novel crystalline coordination polymers with the aim of understanding their structure-property relationships [20,21,22]. In the present work, we intend to explore the possibility of synthesizing novel LnCPs using benzhydrazide (bzz) as a ligand. Herein, we report two new LnCPs, [Sm_2_(bzz)(ben)_6_(H_2_O)_3_]·0.5H_2_O (**1**) and 3D [Eu(bbz)(ben)_3_] (**2**), obtained through in situ synthesis of *N*’-benzoylbenzohydrazide (bbz) and benzoate (ben) ligands from the reactions of bzz and lanthanide nitrate salts under hydrothermal conditions. The in situ ligand formation was clearly evidenced by single crystal X-ray diffraction analysis. Moreover, high-pressure CO_2_ sorption of the activated sample of **1** was investigated in the pressure range up to 50 bar at 298 K. Notably, the room temperature photoluminescence (PL) and lifetime decay behavior of **2** were also examined in the solid state.

## 2. Results and Discussion

### 2.1. Synthesis and Infrared Spectra

**Scheme 1:**
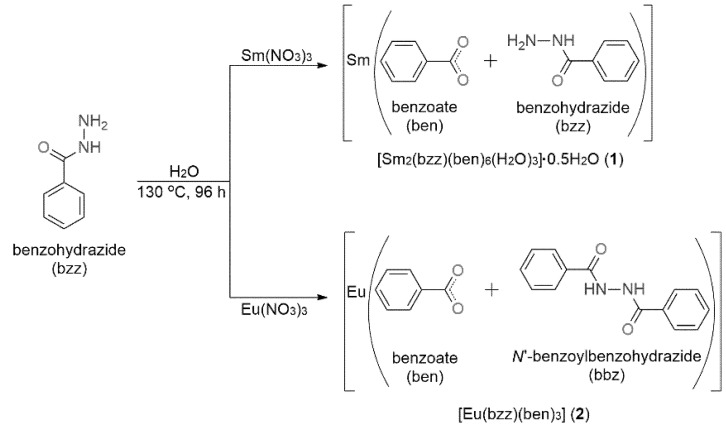
Schematic representation of the in-situ formation of ligands ben and bbz.

As depicted in Scheme 1, compounds **1** and **2** were obtained under the same conditions by the hydrothermal reactions of Ln(NO_3_)_3_·6H_2_O and bzz in a ratio of 1:2 and carried out at 130 °C for 96 h. The new ligands bbz and ben, generated in situ, were formed by the transformation of the bzz precursor. These new ligands can act as bridging and/or chelating ligands for Ln^3+^ ions through oxygen and nitrogen sites to promote the formation of the different polymeric structures viz. 1D chain and 3D network for **1** and **2**, respectively. It should be noted that when the hydrothermal reactions were performed at temperatures above 140 °C, the starting material bzz was completely converted into the ben ligand through the cleavage of C-N bonds [23].

The FT-IR spectra (Figure S1 in the Supplementary Material) of **1** and **2** display characteristic absorption bands arising from the asymmetric and symmetric stretching vibrations of the carboxylate groups in the wavenumber ranges around 1600 and 1400 cm^−1^, supporting the presence of the ben ligand in their crystal structures. The absorption bands in the region 3126–3067 cm^−1^ can be ascribed to N-H stretching vibrations of the hydrazine or hydrazide groups of the ligands. For **1**, the strong and broad absorption bands at 3600–3400 cm^−1^ are assigned as the characteristic absorption bands of O-H stretching of water molecules involved in hydrogen bonding [24]. All features of the FT-IR spectra of both compounds are in accordance with the results of single crystal X-ray diffraction analysis.

### 2.2. Structural Descriptions

**Figure 1:**
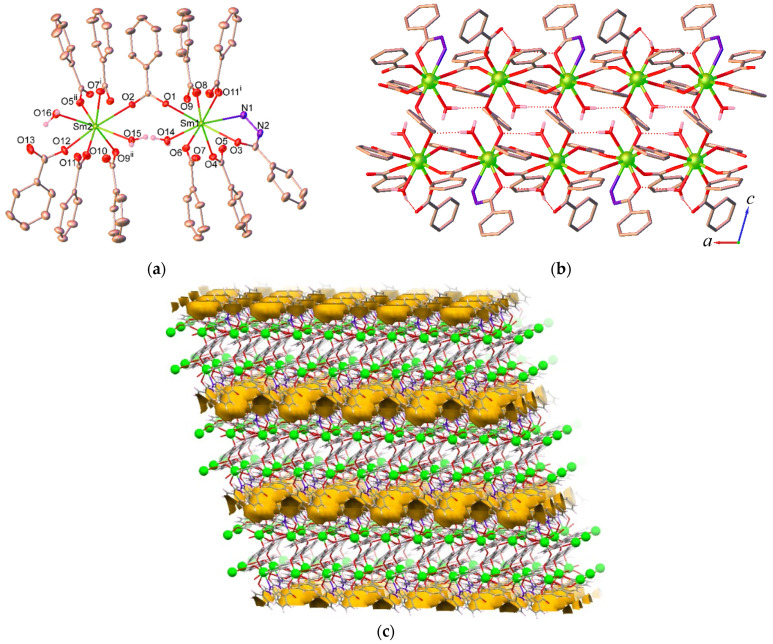
(**a**) Coordination environment of the Sm^3+^ ion in **1**. The thermal ellipsoids are drawn at the 30% probability level for non-hydrogen atoms. Hydrogen atoms attached on the carbon and nitrogen atoms are omitted for clarity. Symmetry codes: (i) −*x*, 1−*y*, 1−*z*; (ii) 1−*x*, 1−*y*, 1−*z*. (**b**) One-dimensional double-chain structure of **1** viewed along the *b* axis. Hydrogen atoms attached on the carbon and nitrogen atoms are omitted for clarity. (**c**) Packing supramolecular structure of **1** viewed approximately along the *b* axis; yellow colored space marks the contact surface of the void spaces.

Compound **1** crystallizes in the triclinic system with the space group *P*-1. As shown in Figure 1a, the asymmetric unit of **1** contains two crystallographically independent Sm^3+^ ions, one bzz ligand, six ben ligands, three coordinated water molecules, and one disordered lattice water molecule with occupancy factors of 0.50. Both Sm^3+^ ions in **1** adopt similar eight-coordinate geometries. Sm1 is coordinated by five oxygen atoms from different ben ligands, one oxygen atom and one nitrogen atom from a bzz ligand, and one oxygen atom from a coordinated water molecule. Sm2 is coordinated by six oxygen atoms from different ben ligands and two oxygen atoms from coordinated water molecules. The coordination polyhedra around the Ln^3+^ metal centers can be best described as distorted trigonal dodecahedral geometries. The Sm-O/N bond lengths in **1** are in the range 2.3088(16)–2.6290(19) Å (Table S2 in the Supplementary Material), which is normal for these types of compounds [25].

The bzz ligand in these compounds exhibits only a bidentate-chelating *η*^2^ mode to bind one Ln^3+^ ion via hydrazide oxygen and nitrogen sites, while the ben ligands adopt both monodentate *η*^2^(O4–O12) and bidentate-bridging *µ*_2_ (O1, O2) modes. As can be seen from Figure 1b, two neighboring Ln^3+^ ions are bridged by carboxylate oxygen atoms of the ben ligands in a *µ*_2_ fashion to form a 1D single chain running parallel to the *a* axis. The Ln···Ln separations through the *µ*_2_-ben ligands in **1** are 5.0195(4) and 5.1423(4) Å. These chains are then cross-linked together in the *c* axis direction via the ben carboxylate oxygen (O1, O2) atoms, giving rise to the formation of a 1D double chain. The Sm···Sm separation between the single chains through the *µ*_2_-ben ligands is 5.9773(4) Å. Further stabilization of these 1D double chains is achieved by extensive O-H···O and O-H···N hydrogen bonds between the coordinated water and amine NH_2_ donors and the carboxylate acceptors along with C-H···π arene edge-to-face interactions. Furthermore, the double chains are interconnected with each other by O-H···O and O-H···N hydrogen bonds involving the lattice water molecules, terminal carboxylate oxygen atoms, and amine NH groups, along with additional N-H···π interactions occurring between bzz molecules. As a result, a 2D sheet is formed which propagates along the *b* axis. The overall 3D supramolecular architecture results from the stacking of the 2D sheets via van der Waals interactions along the *c* axis. The geometrical details of hydrogen bonds for **1** are listed in Table S3 (in the Supplementary Material).

In addition, it is obvious from the crystal structure of **1** described above that the lattice and the coordinated water molecules occupy the intralayer space and the free space between the layers. According to the void analysis performed with the PLATON program [26], using a probe molecule radius of 1.4 Å and a grid interval of 0.2 Å, the simulated solvent-accessible void space of **1** is estimated to be 5.9% (146.0 Å^3^) of the crystal volume after removing the water molecules. The potential void space of **1** calculated in mercury [27] using a probe radius of 1.2 Å is illustrated in Figure 1c.

**Figure 2:**
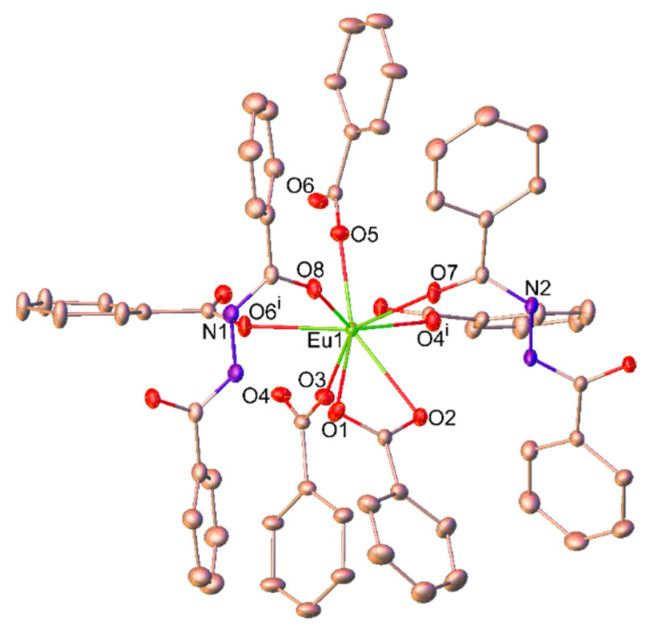
Coordination environment of the Eu^3+^ ion in **2**. The thermal ellipsoids are drawn at the 30% probability level for non-hydrogen atoms. Hydrogen atoms attached on the carbon and nitrogen atoms are omitted for clarity. Symmetry code: (i) 3/2−*x*, 1−*y*, *z*.

For **2**, the hydrothermal in situ synthesis of the new bbz ligand involves the formation of the C-N bond. This ligand is flexible and able to connect two Eu^3+^ centers through the oxygen sites, allowing the generation of a high-dimensional coordination framework. This compound crystallizes in the orthorhombic *Pnna* space group and the asymmetric unit consists of one Eu^3+^ ion, three ben ligands, and a half of two bbz ligands, which lie on an inversion center. As illustrated in Figure 2, the Eu^3+^ ion in **2** is eight-coordinated with six oxygen atoms from five different ben ligands and two oxygen atoms from two bbz ligands, adopting a slightly distorted square antiprismatic geometry. The Eu-O bond lengths range from 2.277(2) to 2.559(2) Å (Table S4 in the Supplementary Material), and are comparable with other reported (EuO_8_) compounds [28].

**Figure 3:**
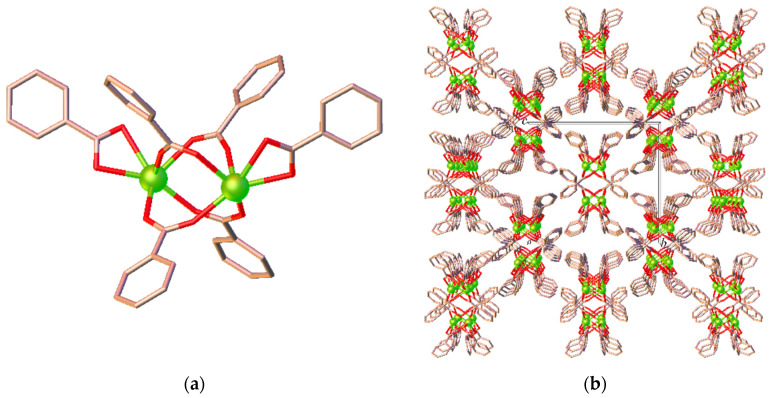

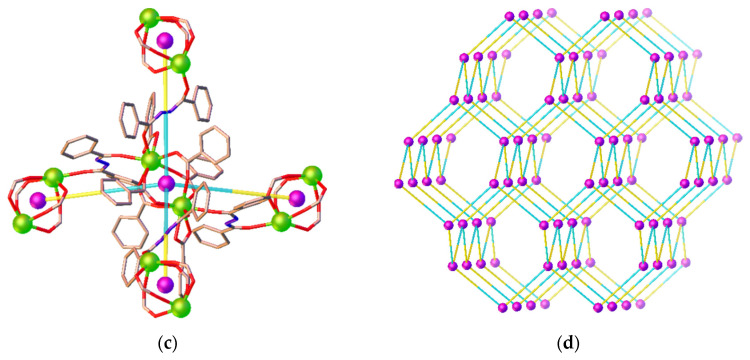
Views of (**a**) the binuclear [Eu_2_(ben)_6_] SBU, (**b**) the packing arrangements of SBUs along *a* axis, (**c**) the simplified 4-connected node of the binuclear SBUs and 2-connected node of the ligand, and (**d**) the a 4-connected (6^6^) **dia** topology in **2**.

Unlike **1**, the ben ligand in **2** adopts two coordination modes *viz*. a bidentate-chelating η^2^ mode (O1, O2) and a bidentate-bridging *µ*_2_ mode (O3–O6). As seen from Figure 3a, two symmetry-related Eu^3+^ ions are bridged by four *µ*_2_-bridging carboxylate groups from four different ben ligands to create a dinuclear [Eu_2_(ben)_6_] secondary building unit (SBU) in which the midpoint of the SBU lies on a twofold rotation axis. The Eu···Eu separation across the dinuclear SBU is 4.2842(4) Å. As depicted in Figure 3b, these SBUs are linked through bbz ligands (not shown) in a *µ*_2_-bridging coordination mode, leading to the formation of a 3D network. The Eu···Eu separations via the bbz ligands are 8.1379(4) and 8.2993(4) Å. The network is further sustained by N-H···O and C-H···O hydrogen bonds that occur between the carboxylate oxygen atoms and the hydrazine NH groups or the phenyl proton of the ligands. In addition, there are also weak C-H···π interactions involving the phenyl rings of the bzz and ben ligands (Table S5 in the Supplementary Material). From a topological point of view, the dimer [Eu_2_(ben)_6_] SBU and the bbz ligand can be regarded as 4- and 2-connected nodes, respectively, as Figure 3c shows. The network structure of **2** could be simplified as a 4-connected (6^6^) **dia** net as depicted in Figure 3d.

### 2.3. PXRD and TGA Analysis

**Figure 4:**
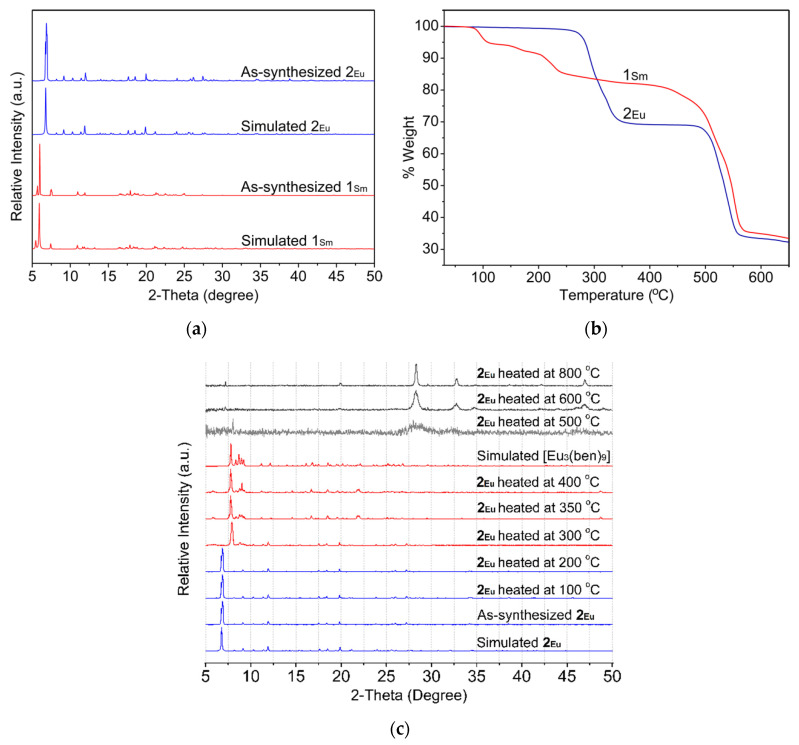
(**a**) Comparison of the experimental PXRD patterns of **1** and **2** with the simulated diffraction patterns from the single crystal X-ray diffraction determination. (**b**) TGA curves of **1** and **2**. (**c**) Variable temperature PXRD patterns of **2**.

The phase purities of **1** and **2** were confirmed by PXRD analysis. As shown in Figure 4a, the experimental PXRD patterns of the bulk samples are in accordance with the simulated patterns derived from crystallographic data, indicating phase purity of the as-synthesized samples. Thermal stabilities of **1** and **2** were also examined by TGA carried out under N_2_ atmosphere from room temperature to 650 °C. The results of this experiment are shown in Figure 4b. For **1**, the first step of weight loss of 5.3% from 50 to 115 °C corresponds to the removal of coordinated and lattice water molecules (calcd 5.1). Thereafter, a weight loss of 11.6%, due to the decomposition of the bzz ligand, occurs in the second step from 150 to 350 °C (calcd 11.5%). The resulting structure is stable up to 425 °C, and then begins to collapse, corresponding to the decomposition of the organic components. Meanwhile, the TGA curve of **2** displays two main steps of weight loss. The first step between 255 and 365 °C, with a weight loss of 30.9%, corresponds to the loss of the bbz molecule (calcd 31.8%). The second weight loss of 38.7% from 470 to 560 °C is attributed to the decomposition of the ben ligands (calcd 48.1%) with the collapse of the skeleton into unidentified products. It is interesting to note that the heated sample of **2** after the first weight loss step in the TGA experiment retained its structural integrity. The resulting material shows significant changes in the PXRD patterns in comparison to the as-synthesized sample, as can be seen in Figure 4c. This could be attributed to the structural changes arising from the removal of the bbz ligands upon heating, which leads to the formation of a new phase. It is clearly visible that four different phases can be recognized in the variable temperature PXRD patterns. As can be seen, the initial phase (identified as the as-synthesized **2**) can be transformed into a second phase when the sample is heated to 300 °C. Clearly, the PXRD pattern of this second phase shows well-defined crystallinity and the main peak positions of the experimental data match quite well with those of the reported 1D polymeric structure of europium-benzoate compound, i.e., [Eu_3_(ben)_9_] [28]. This phase II was stable up to 400 °C, and then underwent phase transformation into an amorphous state (phase III) at 500 °C. Notably, the appearance of new peaks was observed upon further heating from 600 to 800 °C, suggesting a new phase (phase IV) being produced. This possibly results from the pyrolysis of the 1D chain frameworks in air. New sharp diffraction peaks appear at 2θ = 7.23, 19.88, 28.32, 32.79, and 47.01° in the patterns, which is consistent with the pattern of Eu_2_O_3_ (PDF: 00-034-0392). Thus, it can be concluded from the above phenomena that the 3D diamond network of **2** can be converted into the 1D chain structure of [Eu_3_(ben)_9_] through crystalline-to-crystalline transformation upon heating the sample to about 300 °C.

### 2.4. CO_2_ Adsorption Isotherms of ***1***

**Figure 5:**
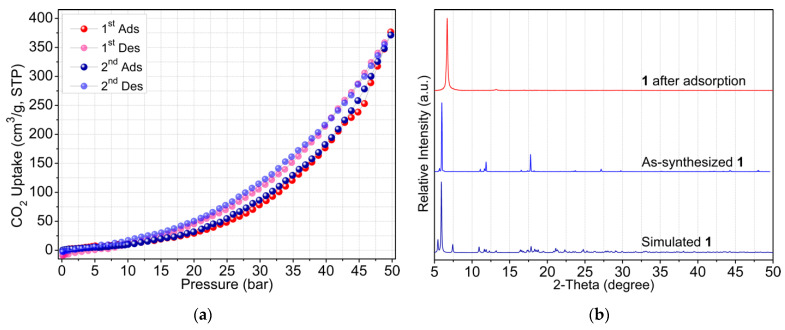
(**a**) Adsorption–desorption isotherms of **1** for CO_2_ at 298 K. (**b**) PXRD patterns of **1** before and after CO_2_ adsorption.

As mentioned above, the crystallographic analysis revealed that compound **1** has a 1D polymeric double chain structure with small void space to accommodate water molecules. On the other hand, **2** exhibits a **dia** topology with dense network structure. Considering its porous structure, the CO_2_ adsorption isotherms of **1,** up to 50 bar, were investigated using a high-pressure volumetric gas adsorption analyzer at 298 K to explore the relationship between structure and adsorption capacity. Prior to the adsorption measurement, the crystalline sample of **1** (~50 mg) was degassed at 110 °C (1 °C/min) under vacuum (2.25 mTorr) for 24 h. As shown in Figure 5a, the activated sample of **1** displayed a type III isotherm according to the IUPAC classification [29], which indicates a weak adsorbent–adsorbate interaction. In the low-pressure region (1–10 bar), the activated sample adsorbed very little CO_2_, which is not surprising as its structure contains a small void space. The amount of adsorbed CO_2_ in **1** increased gradually with increasing gas pressure, which may be related to crystal structure changes including pore expansion and the specific interactions between the CO_2_ sorbate and the sorbent **1**. Apparently, the maximum CO_2_ adsorption value of **1** reached an exceptionally high volumetric uptake capacity of 376.7 cm^3^ g^−1^ (42.5 wt%) at 49.8 bar without saturation. Notably, 1 shows reversible CO_2_ desorption accompanied with a small hysteresis. This observation probably originates from the adsorbent-adsorbate interaction, and this also indicates that the adsorption does not reach equilibrium within the measurement pressure limit of 50 bars.

Furthermore, the CO_2_ adsorption-desorption repeatability of **1** was tested as well. The result demonstrates that **1** can be repeatedly used for at least two consecutive cycles without losing significant adsorption capacity. In addition to this, the PXRD results in Figure 5b show that the crystallinity of **1** can be maintained after two cycles of adsorption. However, the main diffraction peak at 2*θ* ≈ 5.93° was significantly shifted to a higher angle 2*θ* ≈ 6.69° and diffraction peaks at 2*θ* ≈ 11.03°, 11.88°, and 17.73° disappeared, indicating structural change on complete removal of solvent molecules. Regrettably, we failed to determine the crystal structure of the desolvated **1** due to poor diffraction.

### 2.5. Photoluminescence Properies of ***2***

**Figure 6:**
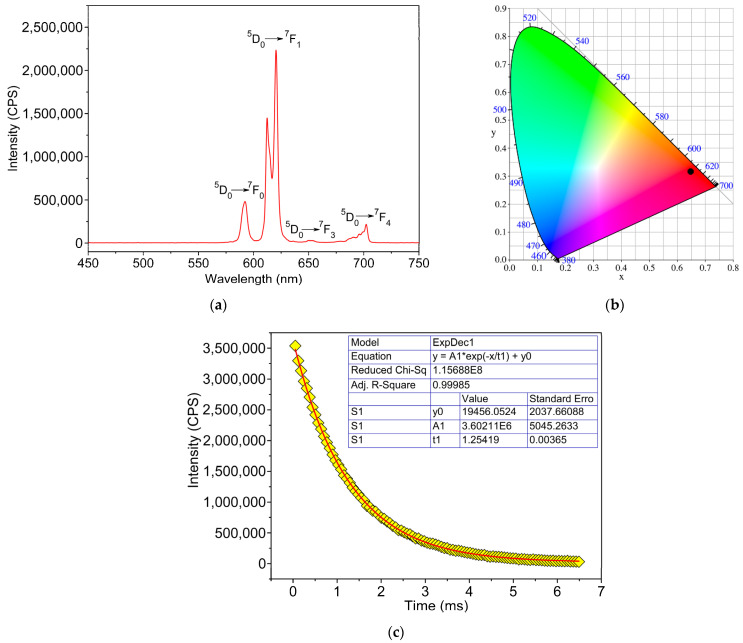
(**a**) Emission spectra and colorimetric coordinates (insert), (**b**) the 1931 CIE chromaticity diagram, and (**c**) emission intensity decay for **2** (*λ*_ex_ = 297 nm).

The solid-state photoluminescence properties and lifetime decay behavior of **2** were investigated at room temperature and the results are displayed in Figure 6. Under excitation at 297 nm (Figure S3 in the Supplementary Material), **2** exhibits the characteristic transitions of Eu^3+^ ion *viz*. ^5^D_0_ → ^7^F_1_ (592 nm), ^5^D_0_ → ^7^F_2_ (612, 620 nm), ^5^D_0_ → ^7^F_3_ (652 nm), and ^5^D_0_ → ^7^F_4_ (702 nm). Among these, the strongest emission peak centered at 620 nm is assigned to the electric dipole transition of ^5^D_0_ → ^7^F_2_, which is hypersensitive to the environment in the vicinity of the Eu^3+^ ions. The strong intensity of this transition is responsible for the bright red emission with CIE coordinates of (0.650, 0.337), while the peak at 590 nm, being second in terms of emission intensity, corresponds to the magnetic dipole ^5^D_0_ → ^7^F_1_ transition, which is insensitive to site symmetry around the Eu^3+^ ion. It can be clearly seen, in the emission spectrum of **2,** that the intensity of the electric dipole transition is stronger than that of the magnetic dipole transition (*I*(^5^D_0_ → ^7^F_2_)/*I*(^5^D_0_ → ^7^F_1_) = 4), which implies that the Eu^3+^ ion resides in a low-symmetry site without an inversion center [30]. Moreover, only one peak is found for the ^5^D_0_ → ^7^F_1_ transition, indicating that there is only one Eu^3+^ ion in the asymmetric unit [31], which agrees with the crystal structure analysis above. Notably, the lifetime for ^5^D_0_ → ^7^F_2_ of Eu^3+^ ion in **2** was calculated to be 1.2 ms. It should be noted that there are no emission peaks observed in **1** upon excitation at various wavelengths from 254 to 420 nm with UV light.

## 3. Experimental Section

### 3.1. Materials and Methods

All starting materials, i.e., Sm(NO_3_)_3_·6H_2_O, Eu(NO_3_)_3_·6H_2_O, and benzhydrazide (bzz) were of reagent-grade quality, and were obtained from commercial sources without further purification. Elemental (C, H, N) analysis was determined with a LECO CHNS 932 elemental analyzer. FT-IR spectra were recorded on a PerkinElmer model Spectrum 100 spectrometer using ATR mode, in the range of 650–4000 cm^−1^. P-XRD measurements were carried out on a Bruker D8 ADVANCE X-ray powder diffractometer using Cu-Kα (*λ* = 1.5418 Å). TGA thermograms were acquired in N_2_ atmosphere on a TGA 55 TA instrument. The measurement was performed from ambient temperature up to 650 °C with a heating rate of 10 °C min^−1^. CO_2_ adsorption isotherms in the pressure range of 0.1–50 bar at 298 K were measured on a Quantachrome iSorb HP1 analyzer. Ultrapure (99.995%) CO_2_ gas was used for the adsorption measurements. The PL spectra and emission decay curves were measured at room temperature using a Horiba Scientific model FluoroMax-4 spectrofluorometer.

### 3.2. Synthesis of [Sm_2_(bzz)(ben)_6_(H_2_O)_3_]·0.5H_2_O *(**1**)*

A mixture of Sm(NO_3_)_3_·6H_2_O (44.7 mg, 0.1 mmol) and bzz (27.3 mg, 0.2 mmol) in distilled H_2_O (4 mL) was placed in a Teflon-lined reactor. The mixture was stirred at room temperature for 30 min, sealed in a 15 mL stainless steel autoclave, and then placed in an oven. The reaction mixture was kept at 130 °C under autogenous pressure for 96 h. After cooling to room temperature, the crystalline product was filtered, washed with distilled H_2_O, and dried in air at room temperature. The product was obtained as light-yellow needle-like crystals with a yield of 84% (37.5 mg) based on Sm(NO_3_)_3_·6H_2_O. Anal. calc. for C_49_H_46_N_2_O_17_Sm_2_: C, 47.63%; H, 3.75%; N, 2.27%. Found: C, 47.54%; H, 3.49%; N, 2.56%. FT-IR (ATR, *ν*/cm^‒1^, s for strong, m for medium, w for weak): 3430(w), 3058(w), 1593(m), 1532(s), 1492(m), 1403(s), 1307(w), 1180(s), 1070(w), 1025(w), 845(w), 712(s), 684(m).

### 3.3. Synthesis of [Eu(bbz)(ben)_3_] *(**2**)*

The procedure was the same as that for **1,** except that Sm(NO_3_)_3_·6H_2_O was replaced by Eu(NO_3_)_3_·6H_2_O (44.9 mg, 0.1 mmol). The product was obtained as light-yellow block- shaped crystals with the yield of 47% (21.1 mg) based on Eu(NO_3_)_3_·6H_2_O. Anal. calc. for C_35_H_27_EuN_2_O_8_: C, 35.66%; H, 3.42%; N, 17.82%. Found: C, 35.43%; H, 3.41%; N, 17.35%. FT-IR (ATR, *ν*/cm^‒1^): 3155 (w), 3055 (w), 3002 (w), 2896 (w), 1643 (m), 1587 (m), 1504 (s), 1477 (s), 1398 (s), 1305 (m), 1173 (w), 1066 (w), 1020 (w), 921 (w), 834 (w), 695 (m).

### 3.4. X-ray Crystallography

Suitable crystals of compounds **1** and **2** were mounted on MiTeGen micromounts using paratone oil (Hampton Research). X-ray diffraction data were collected using a Bruker D8 QUEST CMOS PHOTON II operating at T = 296(2) K. Data were collected using ω and ϕ scans and using Mo-Kα radiation (*λ* = 0.71073 Å). The total number of runs and images was based on the strategy calculation from the program APEX3 and unit cell indexing was refined using SAINT [32]. Data reduction was performed using SAINT and SADABS were used for absorption correction. The integrity of the symmetry was checked using PLATON [26]. The structure was solved with the ShelXT structure solution program using combined Patterson and dual-space recycling methods [33]. The structure was refined by least squares using ShelXL [34] using the OLEX2 [35] interface. In the final refinement cycles, all non-H atoms were refined anisotropically. All C-bound H atoms were placed in calculated positions and refined using a riding-model approximation with C-H = 0.93 Å and *U*_iso_(H) = 1.2 *U*_eq_(C), while all N- and O-bound H atoms were located in a difference Fourier map and refined with N-H = 0.86–0.89 Å and O-H = 0.84 ± 0.01 Å, respectively. The lattice water molecule in **1** presents a positional disorder that was refined as two contributions with a 0.80:0.20 occupancy ratio. The benzene rings of the ligands in **1** and **2** were found to be disordered over two 50% occupancy sites. The details of crystallographic parameters, data collection, and refinements for all complexes are listed in Table S1 in the Supplementary Material.

## 4. Conclusions

Two new mixed ligand LnCPs were synthesized based on in situ-generated ligands formed under similar hydrothermal conditions. The participation of the ben and bbz ligands formed in the transformation of bzz molecules plays an important role in the formation of the structural differences between the network structures of compounds **1** and **2**. Compound **1** shows a 1D double chain, while compound **2** features a 3D network with a 4-connected (6^6^) **dia** topology. Activated compound **1** shows that maximum CO_2_ uptake increases to exceptionally high values of 376.7 cm^3^ g^−1^ (42.5 wt%) under a pressure of 49.8 bar at 298 K with good recyclability. Meanwhile, **2** exhibits a typical red emission in the solid state at ambient temperature with a decay lifetime of 1.2 ms. We believe that the hydrothermal in situ hydrolysis reactions of hydrazide ligand described here will provide an alternative strategy of obtaining novel crystalline LnCPs. Further systematic investigations are currently underway with the aim of exploring various aromatic hydrazide groups containing ligands for hydrothermal in situ self-assembly reactions. We anticipate that this will result in many more novel LnCPs with fascinating structural properties, which will help us to understand the possible reaction mechanism of the in situ ligand synthesis and provide new insights toward the development of crystal engineering of LnCPs.

## Data Availability

The data presented in this study are available in supplementary material.

## Supplementary Materials

The following are available online, Figure S1: FTIR spectra for **1** and **2**, Figure S2: View of C···H-π interactions for **2**, Figure S3: Solid-state excitation spectrum for **2**, Table S1. Summary of crystal data and structure refinement for 1 and 2, Table S2: Selected bond lengths (Å) for **1**, Table S3: Hydrogen-bond geometry (Å, °) for **1**, Table S4: Selected bond lengths (Å) for **2**, Table S5: C···H-π interactions (Å, °) for **2**.

## Sample Availability

Samples of the compounds **1** and **2** are available from the authors.
