# Supplementary material for: Self-Assembly of 1D Double-Chain and 3D Diamondoid Networks of Lanthanide Coordination Polymers through In Situ-Generated Ligands: High-Pressure CO2 Adsorption and Photoluminescence Properties
Source: Molecules. 2021 Jul 22;26(15):4428. doi: 10.3390/molecules26154428 (PMC8347576; doi:10.3390/molecules26154428)
Supplement: Supplementary file 1 [file molecules-26-04428-s001.zip › molecules-1285408-supplementary.pdf]

molecules

Supplementary Information

Self-Assembly of 1D Double-Chain and 3D Diamondoid Networks of Lanthanide Coordination Polymers Through In Situ-Generated Ligands: High-Pressure CO_2 Adsorption and Photoluminescence Properties

Chatphorn Theppitak [1,2], Suwadee Jiajaroen [1,2], Nucharee Chongboriboon [1], Songwuit Chanthee [1], Filip Kielar [3], Winya Dungkaew [4], Mongkol Sukwattanasinitt [5] and Kittipong Chainok [1,*]

[1] Thammasat University Research Unit in Multifunctional Crystalline Materials and Applications (TU-McMa), Faculty of Science and Technology, Thammasat University, Pathum Thani 12121, Thailand; chatphorn.the@dome.tu.ac.th (C.T.), suwadee.jia@dome.tu.ac.th (S.J.); nucharee.cho@dome.tu.ac.th (N.C.); songwuit.c@gmail.com (S.C.)
[2] Department of Chemistry, Faculty of Science and Technology, Thammasat University, Pathum Thani 12121, Thailand
[3] Department of Chemistry, Faculty of Science, Naresuan University, Phitsanulok 65000, Thailand; filipk@nu.ac.th
[4] Department of Chemistry, Faculty of Science, Mahasarakham University, Maha Sarakham 44150, Thailand; winya.d@msu.ac.th
[5] Department of Chemistry, Faculty of Science, Chulalongkorn University, Bangkok 10330, Thailand; mongkol.s@chula.ac.th
* Correspondence: kc@tu.ac.th; Tel.: +66-86-339-5079

Table S1. Summary of crystal data and structure refinement for **1** and **2**.

**Compound**	**1**	**2**
CCDC No.	2083033	2083034
Empirical formula	$C_{49}H_{46}N_2O_{17}Sm_2$	$C_{35}H_{27}EuN_2O_8$
Formula weight	1235.58	755.54
Crystal system	Triclinic	Orthorhombic
Space group	*P*-1	*Pnna*
a (Å)	10.1273(11)	19.1656(5)
b (Å)	15.0479(16)	19.3314(5)
c (Å)	16.9178(17)	17.7307(5)
α (°)	97.949(3)	90
β (°)	105.451(3)	90
γ (°)	91.345(3)	90
V (Å^3)	2456.2(4)	6569.2(3)
Z	2	8
D_{calc} (g cm^{-3})	1.671	1.528
μ (mm^{-1})	2.441	1.960
Crystal size (mm^3)	0.23 × 0.11 × 0.10	0.32 × 0.30 × 0.22
Diffractometer	Bruker D8 QUEST CMOS	Bruker D8 QUEST CMOS
Radiation	MoKα (λ = 0.71073 Å)	MoKα (λ = 0.71073 Å)
Absorption correction	Multi-scan	Multi-scan
θ range for data collection	3.1–26.4°	3.0–30.4°
Data completeness	99.8 (θ_{max} = 26.4°)	99.9 (θ_{max} = 30.4°)
Index ranges	$-12 \leq h \leq 12$	$-25 \leq h \leq 24$
	$-18 \leq k \leq 17$	$-26 \leq k \leq 23$
	$-21 \leq l \leq 21$	$-23 \leq l \leq 23$
Reflections collected	59514	86697
Independent reflections	10102	8483
Observed reflections	8345	6515
R_{int}, R_{sigma}	0.0261, 0.0175	0.0354, 0.0207
Number of restraints	555	710
Number of parameters	823	643
Goodness-of-fit on F^2	1.046	1.046
R_1, wR_2 [$I \geq 2\sigma(I)$]	0.0195, 0.0421	0.0339, 0.0779
R_1, wR_2 [all data]	0.0287, 0.0457	0.0523, 0.0872
$\Delta\rho_{max}$, $\Delta\rho_{min}$ (e Å^{-3})	0.83, –0.41	1.97, –0.37

Table S2. Selected bond lengths (Å) for **1.**

Sm1−O1	2.4505(15)
Sm1−O3	2.5122(15)
Sm1−O4	2.3701(16)
Sm1−O6	2.3088(16)
Sm1−O8	2.3278(17)
Sm1−O1^{i}	2.3498(17)
Sm1−O14	2.4631(18)
Sm1−N1	2.6290(19)
Sm2−O2	2.5470(16)
Sm2−O5^{ii}	2.3220(17)
Sm2−O7^{i}	2.3714(17)
Sm2−O9^{ii}	2.3386(16)
Sm2−O10	2.3551(18)
Sm2−O12	2.4332(17)
Sm2−O15	2.4840(17)
Sm2−O16	2.5279(19)

Symmetry codes: (i) −*x*, −*y*+1, −*z*+1; (ii) −*x*+1, −*y*+1, −*z*+1.

Table S3. Hydrogen-bond geometry (Å,°) for **1.**

D−H···A	D−H	H···A	D···A	D∠H···A
O14−H14A···O2	0.74(3)	2.41(3)	2.945(3)	130(3)
O14−H14A···O9i	0.74(3)	2.29(3)	2.974(3)	155(3)
O14−H14B···O2^{i}	0.79(3)	2.20(3)	2.974(2)	167(3)
O15−H15A···O1^{ii}	0.83(3)	2.11(3)	2.925(2)	164(3)
O15−H15B···O1	0.73(3)	2.39(3)	2.959(2)	136(3)
O15−H15B···O6	0.73(3)	2.42(3)	3.079(2)	151(3)
O16−H16A···O13	0.84(4)	1.81(4)	2.634(3)	168(4)
O16−H16B···O3^{ii}	0.78(3)	2.19(4)	2.972(3)	178(4)
O17−H17B···O13^{iii}	0.85	2.32	2.936(6)	160
O17A−H17C···O13^{i}	0.85	2.32	3.051(5)	143
O17A−H17D···O16^{iii}	0.85	2.32	3.147(5)	162
N1−H1A···O12^{i}	0.89	2.24	3.100(3)	163
N2−H2···O17	0.86	2.06	2.753(5)	138
N2−H2···O17A	0.86	2.16	2.946(5)	153
C14−H14···O17	0.93	2.32	3.176(7)	153

Symmetry codes: (i) −*x*+1, −*y*+1, −*z*+1; (ii) −*x*, −*y*+1, −*z*+1; (iii) *x*, *y*, *z*−1.

Table S4. Selected bond lengths (Å) for **2.**

Eu1−O1	2.475(3)
Eu1−O2	2.559(2)
Eu1−O3	2.277(2)
Eu1−O4^{i}	2.405(2)
Eu1−O5	2.297(2)
Eu1−O6^{i}	2.423(2)
Eu1−O7	2.391(2)
Eu1−O8	2.436(2)

Symmetry codes: (i) −*x*+3/2, −*y*+1, *z*; (ii) −*x*+1, −*y*+1, −*z*+1; (iii) *x*, −*y*+1/2, −*z*+1/2.

Table S5. C···H−π interactions (Å, °) for **2.**

[D−H···ring]	d[X···Cg]	d[X···Plane]	[D−X−Cg]	Symmetry Code
C7−H7···*Cg*2	3.378(19)	3.127(3)	164.6(8)	+*x*, 1/2−*y*, 1/2−*z*
C19−H19···*Cg*1	3.170(7)	2.916(10)	125.4(7)	−1/2+*x*, 1/2−*y*, −1/2+*z*

Cg = centroid of the aromatic ring.

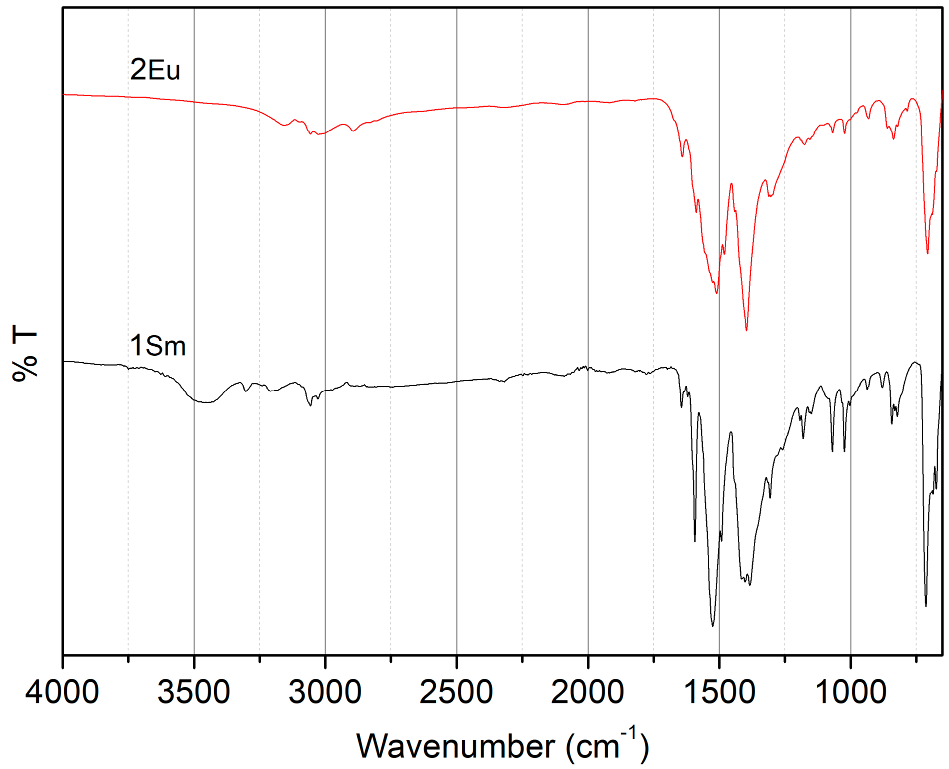

Figure S1. FTIR spectra for **1** and **2**.

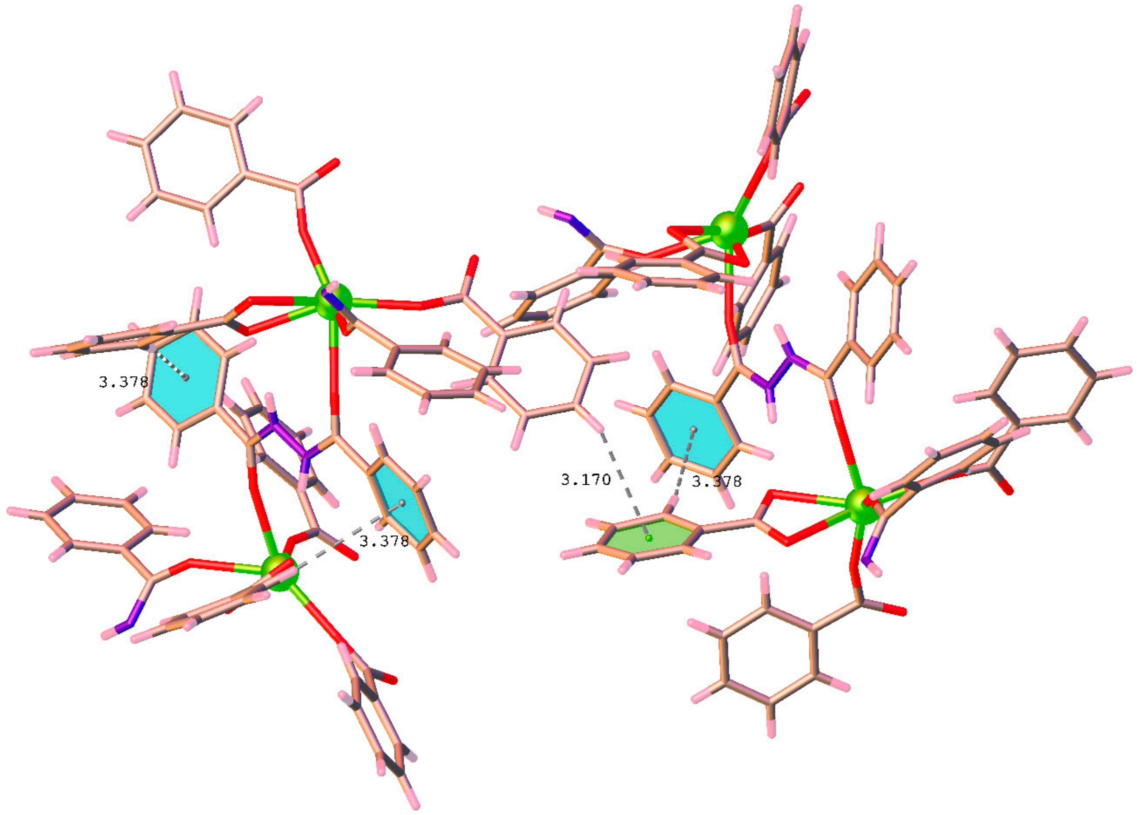

Figure S2. Views of C···H−π interactions for **2**.

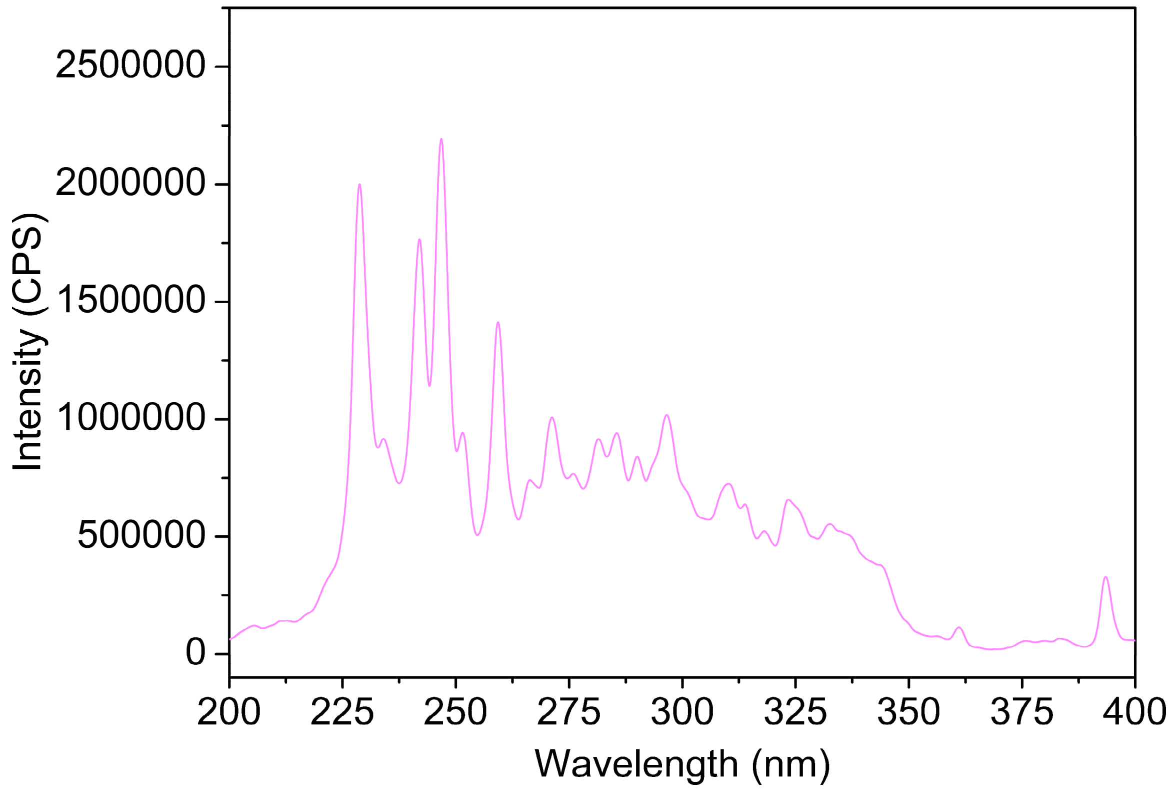

Figure S3. Solid-state excitation spectrum (λ_{em} = 615 nm) for **2**.
